# Caspase 8 Expression Patterns in Meningiomas: A Tissue Microarray Digital Image Analysis

**DOI:** 10.7759/cureus.26182

**Published:** 2022-06-21

**Authors:** Dimitrios Roukas, Anastasios Kouzoupis, Despoina Spyropoulou, Evangelos Tsiambas, Stylianos Mastronikolis, Evangelos Falidas, George Tsouvelas, Vasileios Ragos, Andreas C Lazaris, Nikolaos Kavantzas

**Affiliations:** 1 Department of Psychiatry, 417 Army Equity Fund Hospital (NIMTS), Athens, GRC; 2 Department of Psychiatry, School of Medicine, National and Kapodistrian University of Athens, Athens, GRC; 3 Department of Radiation Oncology, Medical School, University of Patras, Patras, GRC; 4 Department of Cytopathology, 417 Army Equity Fund Hospital (NIMTS), Athens, GRC; 5 Department of Neurosurgery, James Cook University Hospital, Middlesbrough, GBR; 6 Department of Surgery, General Hospital of Halkida General Hospital, Halkida, GRC; 7 Department of Nursing, University of West Attica, Athens, GRC; 8 Department of Maxillofacial Surgery, Medical School, University of Ioannina, Ioannina, GRC; 9 First Department of Pathology, School of Medicine, National and Kapodistrian University of Athens, Laikon General Hospital, Athens, GRC

**Keywords:** tissue microarrays, immunohistochemistry, caspase, apoptosis, meningioma

## Abstract

Background: Caspases (cysteine-aspartic proteases) represent a family of enzymes that critically influence cell homeostasis by being involved in inflammation and apoptosis mechanisms. Meningiomas demonstrate the most common intracranial primary central nervous system tumors in adults worldwide.

Aim: Our purpose was to explore the role of caspase 8 expression in meningiomas’ pathological features.

Materials and methods: A total of 50 meningioma cases were included in the study, comprising a broad spectrum of histopathological sub-types. An immunohistochemistry assay was applied on tissue microarray cores followed by digital image analysis.

Results: Overexpression of caspase 8 protein was observed in 21/50 (42%) cases, whereas the rest of them (29/50, 58%) demonstrated moderate to low levels of the molecule. Caspase 8 overall expression was statistically significantly correlated to grade of the examined tumors and to mitotic index (p=0.001,p=0.002, respectively).

Conclusions: Caspase 8 aberrant expression is observed in meningiomas associated with their differentiation grade and mitotic activity. Targeted therapeutic strategies focused on enhancing caspase 8 expression and also inducing the overall apoptotic activity should be a very promising approach in rationally handling sub-groups of meningioma patients.

## Introduction

Apoptosis corresponds to the genetically programmed variant of cell death mediated by a complex of proteins that influence positively or negatively intrinsic and extrinsic signaling pathways [1]. In cancerous tissues, programmed cell death is inhibited by dysregulated expression of apo- and anti-apoptotic proteins. This genetic imbalance drives the cancer cell to immortalization, inducing aberrant tissue proliferation. For this reason, caspases and other apoptotic mitochondria (or not)-dependent molecules, such as bcl2/bax, are considered important agents for specific targeted therapeutic strategies for enhancing apoptosis rates in malignant cells [2].

Concerning central nervous system (CNS) tumors, meningioma represents the second most common brain tumor and the most common intracranial primary in adults. Interestingly, recurrent dedifferentiated meningiomas are correlated with aggressive biological behaviour. They negatively affect the response rates to surgery/radiation applied therapeutic regimens [3]. Meningiomas’ histological substrate is the arachnoid cap cells of the meninges on the periphery of the brain. Histopathologically, meningiomas comprise a broad spectrum of histopathological sub-types (meningotheliomatous, psammomatus, transitional, fibrous, angiomatous, atypical and anaplastic) [4]. Brain tissue invasion is the most critical histopathological evidence of aggressive biological behavior of the tumor. Furthermore, meningiomas’ extra-cranial metastatic potential and penetration are rare. Some molecular studies have reported - analyzing significant series of meningiomas - a variety of gross chromosomal and specific gene aberrations (rearrangements/intra- or inter-translocations, gains, frame-shift deletions/insertions, point-driver mutations, or in-frame fusions). These imbalances are associated with a progressive differentiation of the corresponding malignancies (Grade I to III) [5,6]. Numerical imbalances affect also other chromosomes besides chromosome 22. Fragment deletions have been detected on chromosomes 1p and 2q33-q35. Regional amplifications occur on chromosome 6p21-p22 and on chromosomes 13q33, 17 and 19. In conjunction with chromosomal and gene instability described above, meningiomas are characterized by a broad spectrum of somatic single nucleotide variants, demonstrated specific single nucleotide polymorphism [7]. Interestingly, there is limited evidence of viral implication in the development of meningiomas, including human cytomegalovirus (HCMV), Epstein-Barr virus (EBV), herpes simplex virus (HSV) 6/7, human papillomavirus (HPV) and hepatitis B virus (HBV) [8]. In the current research study, we explored the role of caspase 8, also called FLICE, expression in meningioma and its potential impact on its specific clinic-pathological features.

## Materials and methods

Study group and tissue specimens

For the purposes of our study, 50 archival, for­malin-fixed and paraffin-embedded meningioma tissue specimens were selected. Broad representation of many meningioma histo-types was a major criterion. According to pathology classification histo-types [9,10], 12 meningotheliomatous, 12 psammomatous, six transitional, five fibrous, two angiomatous, two microcystic, five atypical, five anaplastic and one papillary were recognized. Concerning the corresponding patients, 39 (78%) were female (mean age: 61.5), whereas the rest of them (11, 22%) were males (mean age: 66.2). The ethics committee of the First Department of Pathology, School of Medicine, National and Kapodistrian University of Athens, consented to the use of these tissues for research purposes, according to the World Medical Association Declaration of Helsinki. The tissue samples were fixed in 10% neutral-buffered formalin. Hematoxylin and eosin (H&E)-stained slides of the cor­responding samples were reviewed for confirmation of histopathological diagnoses. All lesions were microscopically diagnosed by an expert pathologist and classified according to the histological typing and grading criteria of the World Health Organization (WHO) including also conventional mitotic indexes (mitoses per high power fields-HPF) [9,10].

Tissue microarrays construction (TMA)

**Figure 1:**
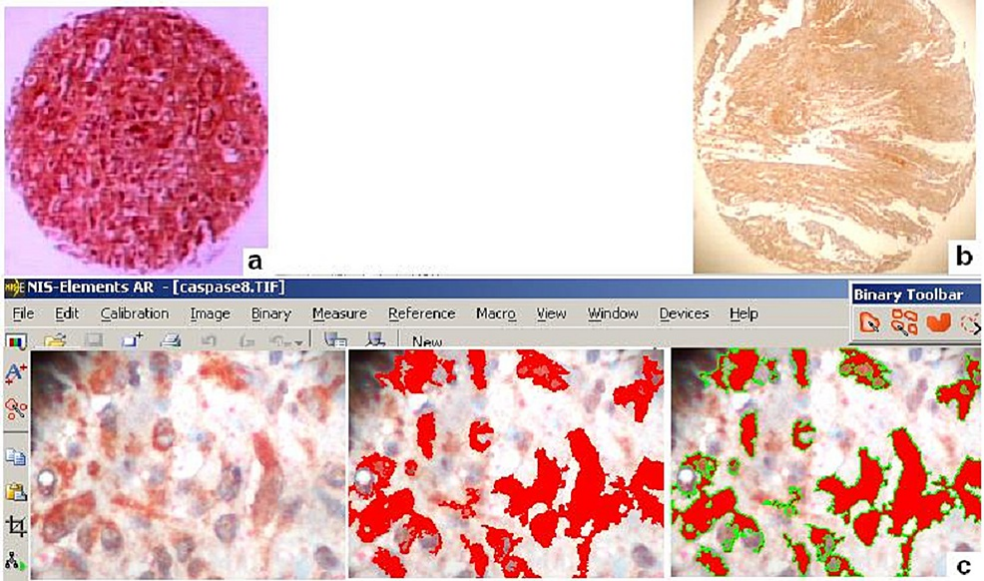
Caspase 8 high expression in a case of meningioma (transitional histo-type) Caspase 8 high expression in a case of meningioma (transitional histo-type) a. A tissue core stained by eosin& haematoxylin (H&E) b. A tissue core immunostained by caspase 8. Note caspase 8 diffuse cytoplasmic and sub-membranous staining pattern c. Digitized evaluation of caspase 8. Red/green areas represent different levels of protein expression as staining intensity values (original magnification 100x, 40x)

Areas of interest were identified on H&E-stained slides by a conventional microscope (BX-50; Olympus, Center Valley, PA, USA). The corresponding paraffin blocks were obtained for the construction of one TMA block using TMArrayer-100 (Chemicon International, Temecula, CA, USA). All of the source blocks were cored and 1.5 mm diameter tissue cylindrical cores obtained from the center of the malignant tissues were transferred from each conventional donor block to the recipient block. The final constructed TMA block contained 50 cylindrical tissue specimens. After 3 mm microtome sectioning and H&E staining, we observed microscopically that the final TMA density was 100% (full tissue microarray core adequacy) (Figure 1a).

Immunohistochemistry assay (IHC)

Ready-to-use anti-caspase 8 liofilisate mouse monoclonal antibody (11Β6; Novocastra, Newcastle, UK) at dilution of 1:30 was applied in the corresponding cases. IHC for the antigen was carried out on a 4μm tissue microarray section. The TMA slide was initially deparaffinized in xylene and rehydrated via graded ethanol. Then the slide was immunostained for the marker according to the EN Vision+ (Dako, Glostrup, Denmark) assay using an automated staining system (I 6000; Biogenex, Fremont, CA, USA) and according to the manufacturer’s instructions. This specific assay is based on a soluble, dextran-polymer system preventing endogenous biotin reaction and increasing the quality of stained slides. Briefly, after peroxidase blocking, the sections were incubated with the primary antibody for 35 min at room temperature and then incubated with horseradish peroxidise-labelled polymer-HRP LP for 30 min. The antigen-antibody reaction was visualized using 3-3, diaminobenzidine tetrahydrochloride (DAB) as a chromogen substrate. Finally, the TMA section was slightly counterstained with hematoxylin for 30 secs, dehydrated and mounted. Breast carcinoma tissues expressing the marker were used as positive controls. For negative controls, the primary antibody was omitted in another slide. Cytoplasmic predominantly and sub-membranous staining was considered acceptable for caspase 8 (Figure 1b).

Digital image analysis assay (DIA)

Caspase 8 protein expression levels were evaluated quantitatively by calculating the corresponding staining intensity levels (densitometry evaluation) in the stained tumor cells. We performed DIA using a semi-automated system (hardware: Microscope CX-31, Olympus; Digital camera, Sony, Tokyo, Japan; Windows XP/NIS-Elements Software AR v3.0, Nikon Corp, Tokyo, Japan). Areas of interest per tissue section were identified (five optical fields at ×400 magnification) and filed in a digital database as snapshots. Measurements were performed by implementing a specific macro (cytoplasmic mainly and partially membranous expression for tumor cells), according to manufacturer’s datasheet. Based on an algorithm, normal tissue sections (control) were measured independently and compared to the corresponding values in malignant tissue sections. A broad spectrum of continuous grey scale values (0-255) at the RedGreenBlue (RGB) analysis was available for discriminating different protein expression levels (Figure 1c). Immunostaining intensity values decreasing to 0 represent a progressive overexpression of the marker, whereas values increasing to 255 show a progressive loss of its staining intensity.

Statistical analysis

For statistical analyses, descriptive and inferential techniques were applied. Statistics software package IBM SPSS v25 (Armonk, NY, USA) was implemented. Quantitative variables were presented as mean ± standard deviation, while the qualitative variables were presented in frequency tables. To evaluate the relationship between qualitative and quantitative variables, cause of the small number of subjects in each group the nonparametric of Mann-Whitney and Kruskall-Wallis was applied. To evaluate the relationship between independent qualitative variables, where appropriate, the control x2 for linear trend and the control of Fisher were applied. Statistical significance (p) was evaluated in pairs and differences < 0.05 were considered statistically significant.

## Results

Total IHC results and statistical differences (p-values) are described in Table 1.

**Table 1:** Clinicopathological parameters and total caspase 8 expression results OE: overexpression (high expression) staining intensity values ≤ 130 at stained cells MLE: moderate-low expression staining intensity values > 130 ≤ 162 at stained cells p-values in bold type refer to statistically significant correlations

Clinicopathological parameters		Caspase 8		p value
Meningiomas (n=50)		OE	MLE	
		21/50 (42%)	29/50 (58%)	
	n (%)	n (%)	n (%)	
Gender				0.316
Male	11 (22%)	6/50 (12%)	5/50 (10%)	
Female	39 (78%)	15/50 (30%)	24/50 (48%)	
Mitotic Index (HPF)				0.002
0-4	33/50 (66%)	8/50 (16%)	25/50 (50%)	
>4>=19	10/50 (20%)	7/50 (14%)	3/50 (6%)	
>=20	7/50 (14%)	6/50 (12%)	1/50 (2%)	
Grade				0.001
I	36 (72%)	9/50 (18%)	27/50 (54%)	
II	8 (16%)	6/50 (12%)	2/50 (4%)	
III	6 (12%)	6/50 (12%)	0/50 (0%)	
Histologic type				0.215
atypical	5/50 (10%)	2/50 (4%)	3/50 (6%)	
anaplastic	5/50 (10%)	3/50 (6%)	2/50 (4%)	
papillary	1/50 (0.5%)	0/50 (0%)	1/50 (2%)	
meningotheliomatous	12/50 (24%)	6/50 (12%)	6/50 (12%)	
psammomatus	12/50 (24%)	5/50 (10%)	7/50 (14%)	
transitional	6/50 (12%)	2/50 (4%)	4/50 (8%)	
fibrous	5/50 (10%)	2/50 (4%)	3/50 (6%)	
angiomatous	2/50(4%)	0/50 (0%)	2/50 (4%)	
microcystic	2/50(4%)	1/50 (2%)	1/50 (2%)	

According to digital image expression analysis, the examined immunostained meningioma tissue microarray cores demonstrated different expression levels of caspase 8. Overexpression of caspase 8 protein was observed in 21/50 (42%) cases, whereas the rest of them (29/50, 58%) demonstrated moderate to low levels of expression. Caspase 8 overall expression was statistically significantly correlated to grade of the examined tumors and to mitotic index (p=0.001, p=0.002, respectively). Interestingly, caspase 8 was not associated with the histologic type of the examined meningioma cases (p=0.215). Additionally, no statistical significance was assessed correlating caspase 8 to gender of the examined patients (p=0.316).

## Discussion

The apoptotic mechanism includes two main pathways: intrinsic and extrinsic. In both of them several proteins are characterized as inducers or inhibitors of apoptosis. The first uses mitochondrial proteins with prominent the cytochrome c from the inter-membrane space of the organelle. Its expression in cytoplasm activates caspases (especially caspase 9) complex under the control of p53 and Bcl-2 (B-cell lymphoma-2) proteins [11,12]. Caspases are significant proteins acting as strong apoptotic death promoters. Caspases (cysteine-aspartic proteases) represent a family of enzymes that influence several functions crucial for cell homeostasis such as inflammation, pyroptosis (a distinct aspect of programmed cell death mediated by microbial infection that triggers an immune response), necroptosis, tissue differentiation and development in the embryonic early stages of life [13]. They also act as tumor suppressor genes, whereas their role in the aging process is under investigation. Approximately, 15 protease proteins have been identified and cloned implicating eight chromosomes (1, 2, 4, 7, 10, 11, 16, and 19). The corresponding protein products are initially inactive (pro-caspases) enzymes. Dimerization or oligomerisation of them creates the final functional heterotetramer domain due to a cleavage process that develops an active heterodimer complex consisting of two units: a small and large one. According to their implication in the apoptotic pathways, caspases are characterized as initiators and executioners, respectively. In the first group are caspase 2, 8, 9, and 10, whereas caspase 3, 6, and 7 belong to the second category [14].

In the current study we analyzed by IHC a significant number of meningioma tissue cores (TMA) including a variety of histological sub-types and grades. Although TMAs provide a reliable tissue substrate for a multiple-case co-analysis, there are some limitations regarding the quantity of the corresponding tissue mass on the slides. We also selected a broad spectrum of meningioma variants for the construction of the TMA. Although this is an advantage for the study, the extended stratification of the cases affects partially the statistical analysis. Caspase 8 expression was observed in high, moderate, and low levels associated with differentiation grade and mitotic activity of the examined malignancies, but not with meningioma-specific histo-types. In fact, caspase 8 overexpression demonstrates a stratification in different grades and mitotic indexes in the examined tissue cores. In these cases, enhancement of the apoptotic mechanism may be combined with other apoptotic molecules' overexpression. A study group analyzing a series of meningiomas reported high expression levels of tumor necrosis factor-related apoptosis-inducing ligand R2 (TRAIL-R2) combined with low levels of caspase 8 [15]. Additionally, another protein and gene expression analysis focused on apoptotic pathways including c-FLIP, XIAP, Bcl-2, caspase 3, 8 and 9, cytochrome c, APAF 1 and Smac/DIABLO molecules reported low protein levels regarding caspases. The study group also identified a potential blocking of these apoptotic inducers mediated by c-FLIP inhibition on them [16]. Interestingly, specific polymorphisms affecting genes such as caspase 8, NF2, XRCC1, and BRIP1 are related to increased risk for meningioma rise [17]. In contrast, there are controversial results regarding the role of a specific polymorphism in meningioma tissues. A study group reported potential involvement of CASP8 polymorphism D302H, whereas another one showed no association with increased risk for meningioma development [18,19]. Additionally, CASP8 Ex13+51G>C variant seems to influence positively the meningioma development risk [20]. Concerning aberrant methylation in caspase 8, there is no evidence that this mechanism is present in meningiomas [21].

Enhancement of apoptotic rates in solid malignancies, including meningioma, is a critical issue for applying chemo-targeted therapeutic strategies. According to some studies, there are new, most promising agents for inducing caspases' and other apoptotic molecules' activity. One of them is fenretinide. It is a synthetic retinoid that increases apoptotic rates in tumor cell cultures in several malignancies. A study group reported significant levels of caspase activation in meningioma mediated by fenretinide. Interestingly, the agent provided apoptosis in all three grades of meningioma primary cells cultures [22]. Another molecule under investigation is the ASA404 (DMXAA). It acts as a vascular disrupting agent involved indirectly in apoptosis. ASA404 induced cleaved caspase 8 activity and inhibited the proliferation and growth of tumors in cell lines [23]. Additionally, valproic acid (VPA), a commonly used anti-epileptic drug, seems to induce apoptosis by increasing cleaved caspase 3 and PARP apoptotic molecules in meningiomas’ stem cells cultures providing also elevated radio-sensitivity to them [24]. Finally, because caspase 8 is a member of apoptotic proteins, interactions with other ones - including caspase 3 and calpain - are crucial for targeting the proliferation/apoptosis equilibrium in brain malignancies [25].

## Conclusions

In conclusion, although caspase 8 expression demonstrates different expression patterns in meningiomas, it is associated with differentiation grade and mitotic activity in them. Our findings showed that alterations of the molecule at the protein level are critical for the biological behavior of the malignancy. Further studies analyzing the molecule at the gene level should provide novel data explaining these protein expression patterns. Additionally, interactions with other caspases or calpain should be a target for understanding apoptosis in brain malignancies. Based on these basic research studies, a variety of agents have been already developed and experimentally applied as potential novel therapeutic strategies in meningioma for enhancing caspase-mediated apoptotic death and response rates to specific chemo-radiation regimens. Implementation of sophisticated techniques, such as DIA assays, are useful tools in modern cyto-pathology improving the accuracy and objectivity in evaluating protein expression levels and patterns compared to conventional eye-based microscopy. Concerning our study, expert pathologist's eye-based IHC evaluation was closely related with DIA measurements. But, the human eye is very limited in distinguishing the exact staining intensity levels of protein expression in tissue and cytological slides.
